# Testosterone is associated with cooperation during intergroup competition by enhancing parochial altruism

**DOI:** 10.3389/fnins.2015.00183

**Published:** 2015-06-12

**Authors:** Luise Reimers, Esther K. Diekhof

**Affiliations:** Neuroendocrinology Unit, Biocenter Grindel and Zoological Museum, Institute for Human Biology, University of HamburgHamburg, Germany

**Keywords:** testosterone, intergroup conflict, prisoner's dilemma, altruistic behavior, competition

## Abstract

The steroid hormone testosterone is widely associated with negative behavioral effects, such as aggression or dominance. However, recent studies applying economic exchange tasks revealed conflicting results. While some point to a prosocial effect of testosterone by increasing altruistic behavior, others report that testosterone promotes antisocial tendencies. Taking into account additional factors such as parochial altruism (i.e., ingroup favoritism and outgroup hostility) might help to explain this contradiction. First evidence for a link between testosterone and parochial altruism comes from recently reported data of male soccer fans playing the ultimatum game. In this study high levels of endogenous testosterone predicted increased altruistic punishment during outgroup interactions and at the same time heightened ingroup generosity. Here, we report findings of another experimental task, the prisoner's dilemma, applied in the same context to examine the role of testosterone on parochial tendencies in terms of cooperation. In this task, 50 male soccer fans were asked to decide whether or not they wanted to cooperate with partners marked as either fans of the subject's own favorite team (ingroup) or fans of other teams (outgroups). Our results show that high testosterone levels were associated with increased ingroup cooperation during intergroup competition. In addition, subjects displaying a high degree of parochialism during intergroup competition had significantly higher levels of testosterone than subjects who did not differentiate much between the different groups. In sum, the present data demonstrate that the behavioral effects of testosterone are not limited to aggressive and selfish tendencies but may imply prosocial aspects depending on the context. By this means, our results support the previously reported findings on testosterone-dependent intergroup bias and indicate that this social hormone might be an important factor driving parochial altruism.

## Introduction

The steroid hormone testosterone is known to play an important role in modulating human behavior, especially during social interaction. During the past, testosterone has been widely associated with aggressive and dominant behavior, a view that is mainly based on animal studies or correlational evidence in humans linking endogenous testosterone levels to self-reports or personality scales on aggressive and antisocial behavior (Mazur and Booth, 1998; Archer, 2006). More recently, researchers have begun to further investigate the effects of testosterone on human behavior in social contexts by applying economic decisions paradigms adapted from game theory such as the ultimatum game or the prisoner's dilemma. These paradigms allow for a direct measure of aggressive or selfish behavior under laboratory conditions, which can then be linked to habitual testosterone levels. To date, studies from this context revealed inconsistent results with some suggesting that testosterone promotes prosocial behavior such as increased altruistic punishment (i.e., bearing personal costs for sanctioning selfish behavior and violations of social norms) or fairness (Burnham, 2007; Eisenegger et al., 2010; Mehta and Beer, 2010), whilst others report a positive association between testosterone and antisocial tendencies, for instance in the form of decreased generosity (Zak et al., 2009). In addition to these conflicting results, other researchers did not find any behavioral effects of testosterone during social exchange tasks (Zethraeus et al., 2009) or observed both, anti- and prosocial influences, in decision contexts with or without the possibility of financial betrayal, respectively (Boksem et al., 2013). Important to note are the methodological differences between the above mentioned studies. While some examined the effects of endogenous testosterone levels (Burnham, 2007; Mehta and Beer, 2010) others administered testosterone (Zak et al., 2009; Zethraeus et al., 2009; Eisenegger et al., 2010; Boksem et al., 2013). Additionally, some studies investigated effects in both sexes (Mehta and Beer, 2010), whereas others only tested men (Zak et al., 2009) or females (Eisenegger et al., 2010; Boksem et al., 2013). One study even tested postmenopausal women (Zethraeus et al., 2009).

Another possible explanation for these controversial findings might be that the assumption of a direct link between testosterone and aggressive or prosocial behavior is oversimplifying a rather complex relationship. Taking into account additional factors might help to gain a better understanding of the mechanism by which testosterone shapes human behavior. For instance, group membership and social closeness have been shown to influence altruistic punishment in that ingroup members are protected more often than outgroup members even if this implies personal costs (e.g., Bernhard et al., 2006; Baumgartner et al., 2012; Goette et al., 2012). Preferential treatment of ingroup members and increased hostility toward the outgroup, even at one's own cost, are common human behaviors and have been referred to as parochial altruism (Choi and Bowles, 2007; Bowles, 2009; García and van den Bergh, 2011). A second important aspect is intergroup competition. Several studies have shown that the context of an intergroup competition alters altruistic behavior compared to an individual setting. Rebers and Koopmans (2012) assigned subjects to groups and conducted a version of the n-person prisoner's dilemma that included an option to punish defectors of the own group. They observed more altruistic punishment when the different groups were competing with each other than during a context with no intergroup competition. Other studies examined the effect of intergroup competition using real social groups. For instance, Van Vugt et al. (2007) found that male university students cooperated more with their own group (i.e., fellow students) during an intergroup competition against students from other universities than in an individual setting without group competition. Another study investigated the tendency for cooperation between members of different Swiss Army Platoons (Goette et al., 2012). Results showed that ingroup favoritism and outgroup hostility increased in a group competition between the different Platoons compared to a neutral context, during which subjects also faced counterparts from the different Platoons but played individually for their own payoff. There are also findings from other contexts, such as cognitive tasks, indicating an effect of group competition on the link between testosterone and task performance (Mehta et al., 2009), which suggest that testosterone effects may depend on the type of social challenge (i.e., individual vs. intergroup competition). In addition, there exists a large body of literature on the influence of testosterone levels on behavior during competition. It has been shown repeatedly that testosterone levels rise after winning a competition and that high testosterone levels are associated with competitive drive and the willingness to engage in competitions (for review please see Mazur and Booth, 1998; Archer, 2006; Carré and Olmstead, 2015).

But what leads to assume that parochial altruism and intergroup competition might explain the contradicting results considering the behavioral effects of testosterone during social interaction? According to a recently proposed theory, the “male warrior hypothesis,” men are more prone to form coalitions, engage in intergroup conflicts and they display increased altruistic tendencies in the presence of an intergroup competition (Van Vugt et al., 2007; McDonald et al., 2012). Since testosterone is the predominant hormone in men, it might be involved in the modulation of these parochial patterns, thereby also accounting for individual behavioral differences. Based on this assumption, testosterone might enhance different types of behavior depending on the situation (individual vs. competition context) and interaction (own group vs. other group) rather than being restricted to promote either aggressive or altruistic behavior.

Initial evidence for a testosterone-driven modulation of parochial altruism comes from recently published data of male soccer fans playing a single-shot version of the ultimatum game (UG) (Diekhof et al., 2014). In the UG two players interact: the proposer has to offer a share of an initially endowed sum of money or points to the responder. The responder can then decide whether or not to accept this offer (which can vary in terms of fairness). In case of rejection, both players receive nothing. In this study subjects played in the role of the responder and interacted once with different proposers, who were either marked as fans of the subject's own favorite team (i.e., ingroup) or as fans of other teams of different rivalry (i.e., outgroups). The group identities and the offers of the proposers were predetermined by the experimental protocol, but subjects were led to believe that they faced real decisions of former participants. In addition, the UG was played in two different contexts: a neutral session and a competition between the groups composed of fans of the same team. Furthermore, subjects were also asked to switch to the role of the proposer and offer a share of 10 points to an ingroup member and members of the three outgroups. Regarding the proposals there was no differentiation between a neutral or competitive context and subjects only made one offer to each group. Findings indicate that bargaining behavior is highly influenced by social distance as well as the context. Furthermore, endogenous testosterone was associated with a pronounced degree of parochialism. In the competition context individuals with higher testosterone levels rejected offers by fans of rivaling soccer teams more often. At the same time, high testosterone levels predicted higher and thus more generous offers to ingroup members.

However, norm-compliant proposals in the UG may not capture true altruistic behavior entirely free from selfish motives since the probability of rejection and thus financial loss decreases with higher offers. Therefore, here we report results of a second game paradigm that was conducted in this study cohort. Subjects also performed a version of the prisoner's dilemma (PD), during which they had to decide whether or not they wanted to cooperate with another soccer fan. In the PD two players are asked simultaneously if they want to cooperate with each other or not. If only one player chooses to cooperate, the other one receives maximum payoff, which makes defection (i.e., no cooperation) the preferable strategy from an economic perspective. Nonetheless, it has been repeatedly observed that humans display a tendency toward cooperation (Camerer, 2003; Fehr and Fischbacher, 2003). Here, the PD was applied to test whether endogenous testosterone levels are also linked to prosocial behavior in terms of cooperation, which would confirm the previously observed positive effect on altruistic punishment in the UG. Similarly to the proposer role in the UG, the PD implies a trade-off between personal payoff and expectations on the behavior of the opponent, which will affect the final outcome. However, making an offer in the UG presumably requires even more complex considerations since the expectations on the reactions of the responders might also vary with the different options for proposals that can range between one and five points. In contrast to that, the PD only leaves two options, cooperation or defection, which simplifies the process of weighing up selfish motives against predicted reactions of the opponent. Consequently, the decision to cooperate in the PD might be less ambiguous in terms of a financial trade-off than offering a high share in the UG. Hence, the PD was additionally performed to obtain more evidence complementing the positive association between testosterone and prosocial behavior toward ingroup members in the UG (Diekhof et al., 2014). In accordance with the procedure described in Diekhof et al. (2014) this task was played in two contexts: the neutral setting and the group competition.

We hypothesized that cooperation rates would decrease with increasing social distance to the opponent and that this group-dependent behavior would further escalate in the competition context. Individuals with high testosterone were predicted to show increased ingroup cooperation in combination with decreased outgroup cooperation (i.e., parochial altruism), especially during the intergroup competition.

## Materials and methods

### Participants

50 healthy male soccer fans (mean age ± SD: 24.6 ± 3.5) participated in this study, which was approved by the ethics committee of the Medical Council of Hamburg (Aerztekammer Hamburg). Participants were recruited among students of the University of Hamburg via online advertisement and flyers. They were told that they could win up to 15 Euros depending on their outcome during the PD and the UG (results from the latter are reported in Diekhof et al., 2014). All subjects were healthy and reported neither use of medication nor alcohol or drug abuse. Prior to testing, subjects were asked about their general interest in soccer via questionnaire to ensure a strong feeling of group affiliation. This questionnaire included a rating of the question “How much are you interested in soccer?” on a 5-point-Likert-scale as well as questions considering stadium attendance or fan merchandise. Subjects also had to rate all teams of the German Premier League (Bundesliga) as well as one local soccer team of the second division according to their own preferences on a 5-point-Likert-scale ranging from 1 (“my favorite team”) to 5 (“my least favorite team”). This rating was then used to assign individualized “fan identities” to the presented opponents in the PD, so that subjects encountered either fans of their own favorite team or fans of other teams of varying rivalry. Inclusion criteria for this study implied that one soccer team was rated as the favorite team (score of 1), another team as the least favorite (score of 5), and that subjects also considered at least one team as “neutral” (score of 3). Written informed consent was obtained from all participants before the experiment.

### Experimental design

**Figure 1:**
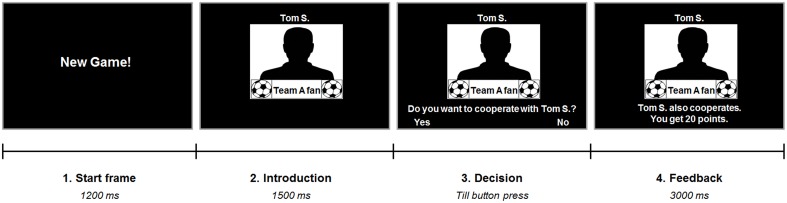
**Experimental paradigm**. Each trial started with a start frame informing the subject that now there will be a new interaction. Next, subjects saw a male silhouette representing the second player along with two small soccer team logos as well as the written name of the team to indicate the second player's favorite team. The first name and initial of the last name of the opponent was presented to increase plausibility of a real person. After this, subjects were asked to decide whether or not they would like to cooperate with the opposing player. They indicated their response via right or left button press. The second player's decision was then revealed along with feedback on the outcome according to the subject's decision.

Participants performed a version of the PD with 40 single-shot interactions. A repeated version was used to create a more realistic social setting (e.g., Axelrod and Hamilton, 1981) implying several encounters with the same team. They were told that during the experiment they would interact with other soccer fans, who were tested earlier, and be presented with their former decisions. In fact, the decisions and fan identities of the opposing players were predetermined to test subjects' behavior in four different conditions: interactions with (a) fans of the subject's own favorite soccer team (ingroup), (b) fans of the most disliked soccer team (antagonistic outgroup), (c) fans of a soccer team that was rated as neutral by the subject (neutral outgroup), and (d) fans of an unknown cricket team (unknown outgroup). Hence, the teams in the first three different conditions were selected individually according to the participant's prior preference rating. At the beginning of each round in the game both players were endowed with 20 points. If the two players decided to cooperate, both received 40 points. In case of defection (no cooperation) at both sides, the two players both kept their initial 20 points. Maximum payoff, however, could be won if one player decided to keep his points whilst the other cooperated. In this case the defector received 60 points and the other player got nothing. Participants were told that their achieved sum of points over all interactions would later be converted to real money, but the exact conversion factor for points to Euros was not given in order to prevent the decision making progress being disturbed by concurrent computing. Each of the four conditions was represented by ten trials, of which three involved defection by the other player. Trials were presented in pseudorandomized order and counterbalanced for condition transitions. Each trial began with a start frame indicating a new interaction. After this, participants were shown a male silhouette with small team logos and the written team name beneath it representing the second player. The first name as well as the last name's initial of the in actual fact fictive second player were also presented to increase authenticity of the game and emphasize the social nature of the task (for a similar approach see Sanfey et al., 2003). Next, participants were asked whether or not they would like to cooperate with this person and to indicate their decision via right or left button press. After this, feedback on the second player's decision and the outcome was given (Figure 1). The PD was played in two different contexts: during the first session, participants were told to maximize their own outcome (neutral context). In the second session, however, they were instructed that they could win extra points if their own team, which included all fans of the same soccer team, would finally outperform the other teams (competition context). Consequently, in this session participants have to reduce selfish impulses in interactions with ingroup members (i.e., choosing to cooperate instead of defecting) to ensure maximum payoff. Again, we refrained from informing the subjects about the exact amount of extra points to be won during the competition. This was done for similar reasons as with the conversion factor. In fact, the extra reward of the PD competition context constituted 20% of the total points that could be won during the whole experiment. Notably, subjects neither asked for the extra sum of points nor the conversion to money. Written instructions were given before both sessions (see Supplementary Material) and a short training version was conducted before the start of the real session. In both sessions participants also completed a version of the UG (for results see Diekhof et al., 2014). The order of the two games was counterbalanced across participants, but the neutral sessions were always completed first.

### Saliva samples and assays

Participants provided five saliva samples over a period of 2 h in the morning of the test day to determine salivary concentrations of free testosterone. Sampling began at home directly after waking up and continued with an interval of 30 min to ensure a representative sample controlling for highly variable concentrations due to fluctuating secretion patterns. During collection subjects were instructed to refrain from eating, smoking, chewing gum, and drinking anything besides water. Tooth brushing was allowed after the first sample, but not immediately before collecting the second. Samples were collected in 2 ml polypropylene Eppendorf tubes and frozen at −20°C until further analysis. Before assaying, all samples were thawed and mixed by vortex and centrifuged at RCF 604 × g for 5 min (i.e., 3000 rpm in a common Eppendorf Minispin centrifuge) to separate saliva from mucins and other residuals. Aliquots were prepared by mixing equal volumes of each of the five samples. Samples that were not clear and colorless were left out to exclude blood contaminated saliva. Therefore, some aliquots contained saliva of less than five samples. Salivary concentration of free testosterone was assessed using an enzyme-linked immunosorbent assay (ELISA) kit by Demeditec Diagnostics with a sensitivity of 2.2 pg/ml (denoted intra-assay coefficients of variation: 6.58% at 90.8 pg/ml, inter-assay variation: 7.4% at 74.3 pg/ml). All samples were assayed twice and two control samples (low and high) were also added. Two assay kits were used since the sample size extended assay space.

### Statistical analyses

All statistical analyses were performed with SPSS 19. First, mean cooperation rates for each participant in each condition were determined. One subject had to be excluded from further analyses due to a technical error, which prevented the completion of the second experimental session. Repeated-measures ANOVA was used to test for an effect or interaction of the factors “team” and “context” on the cooperation rates. Wilcoxon-rank tests were conducted as post hoc comparisons. To identify possible associations between testosterone and cooperation rates Spearman rank correlations were used. Furthermore, testosterone levels were compared between subjects displaying a high or low parochial pattern with independent *t*-Tests. For this purpose, the ingroup bias for each subject was determined by calculating the difference between the cooperation rates with the ingroup and the antagonistic outgroup during the competition. Accordingly, a high value of ingroup bias indicated more cooperation with the ingroup relative to the antagonistic outgroup, whereas a low value represented the opposite. Median-split was then used to divide the sample in two groups: subjects with an ingroup bias above the median of 90% (i.e., the “parochialists,” *n* = 23; all subjects in this group had an ingroup bias of 100%) and subjects below the median (i.e., the “individualists,” *n* = 20; ingroup bias [mean ± sem]: 43.00 ± 7.54%). Significances are reported two-tailed if not otherwise indicated and one-tailed in case of directed a priori hypotheses.

## Results

**Figure 2:**
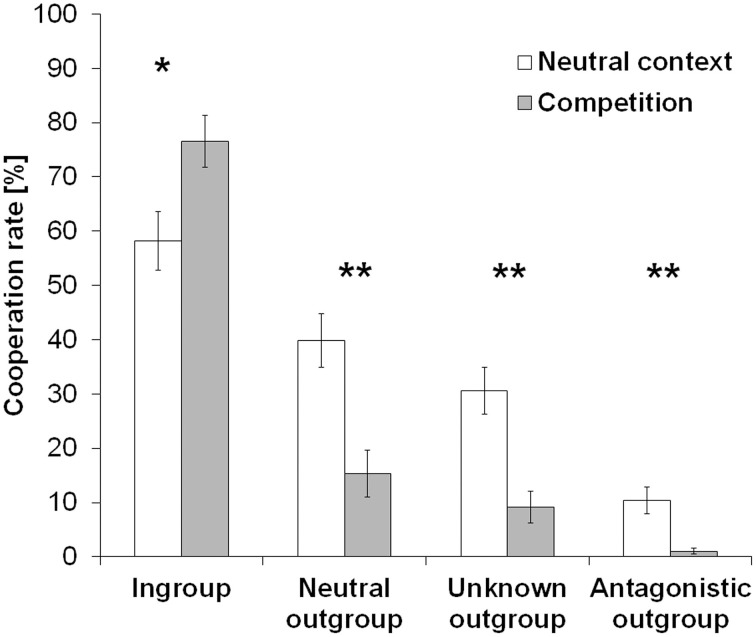
**Cooperation rates in the prisoner's dilemma**. Cooperation rates decreased with increasing social distance to the opposing player's team. The group competition context further accentuated this parochial pattern resulting in increased cooperation rates with ingroup members whereas outgroup cooperation decreased (^*^p < 0.01, ^**^p < 0.001). Error bars indicate standard errors from mean (SEM).

First, we investigated the effect of group membership and context on cooperative behavior. A 4 (team: ingroup, neutral outgroup, unknown outgroup, antagonistic outgroup) × 2 (context: neutral session, competition) repeated-measures ANOVA revealed highly significant effects for context [*F*_(1, 48)_ = 12.69, *p* = 0.001, η^2^_*p*_ = 0.21] and team [*F*_(3, 144)_ = 85.22, *p* < 0.001, η^2^*_p_* = 0.64] as well as an interaction between the factors team and context [*F*_(3, 144)_ = 23.40, *p* < 0.001, η^2^*_p_* = 0.33]. *Post-hoc* Wilcoxon signed-rank tests showed that cooperation rates were lower in the competitive context than during the neutral session (*Z* = −3.58, *p* < 0.001, *n* = 49; cooperation rate [mean ± sem]: neutral session = 34.76 ± 3.19%, competition = 25.52 ± 2.10%). Further, cooperation rates increased with increasing social distance resulting in significant differences between the cooperation with the different teams except for the comparison between the neutral and the unknown team, which only reached statistical trend level (*Z* = −5.85, *p* = 0.97, *n* = 49). The “team” × “context” interaction was mainly accounted for by significant higher cooperation rates with ingroup members during the competition than during the neutral session (*Z* = −3.03, *p* = 0.002, *n* = 49) and significantly lower cooperation rates with neutral, unknown, and antagonistic outgroup during the competition than during the neutral session (neutral outgroup: *Z* = −4.33, *p* < 0.001; unknown outgroup: *Z* = −4.69, *p* < 0.001; antagonistic outgroup: *Z* = −3.50, *p* < 0.001; *n* = 49). Figure 2 shows mean cooperation rates with all teams in both sessions. In addition, Table 1 lists all mean cooperation rates as well as the behavioral change in cooperation rates during the competition as compared to the neutral context (Δcontext = cooperation rate competition—cooperation rate neutral session).

**Table 1:** **Cooperation rates in the prisoner's dilemma**.

**Team**		**Mean cooperation rate [%] ± SEM**		
	**Sample**	**Neutral session**	**Competition**	**Contextual difference of cooperation rates (Δcontext: competitive—neutral session)**
Ingroup	All:	58.16 ± 5.46	76.55 ± 4.84	18.39 ± 5.49
	Parochialists:	66.96 ± 8.35	100.00 ± 0.00	33.04 ± 8.35
	Individualists:	46.00 ± 8.22	45.55 ± 7.65	−0.45 ± 7.12
Neutral outgroup	All:	39.86 ± 5.02	15.31 ± 4.34	−24.55 ± 5.09
	Parochialists:	54.78 ± 8,24	25.65 ± 8.52	−29.13 ± 9.62
	Individualists:	22.65 ± 5.86	6.00 ± 2.34	−16.65 ± 5.27
Unknown outgroup	All:	30.61 ± 4.33	9.18 ± 2.99	−21.43 ± 3.58
	Parochialists:	32.17 ± 6.69	10.00 ± 4.87	−22.17 ± 5.90
	Individualists:	25.00 ± 6.18	6.00 ± 3.03	−19.00 ± 5.47
Antagonistic outgroup	All:	10.41 ± 2.54	1.04 ± 0.53	−9.37 ± 2.65
	Parochialists:	12.61 ± 4.76	0.00 ± 0.00	−12.61 ± 4.76
	Individualists:	9.50 ± 2.85	2.55 ± 1.25	−6.90 ± 3.35

*Means and standard errors (SEM) for the different experimental conditions (team and context) for all participants (n = 49) and for individuals displaying a high ingroup bias during the competition (“parochialists,” n = 23) or a low ingroup bias (“individualists,” n = 20)*.

**Figure 3:**
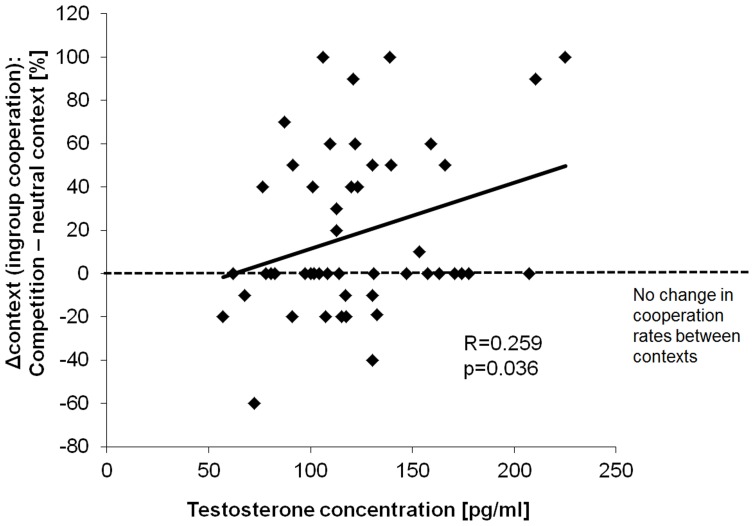
**Effect of testosterone on ingroup cooperation**. High testosterone levels were associated with increased ingroup cooperation during the group competition relative to the neutral context (Δcontext: cooperation rates competition—neutral context).

**Figure 4:**
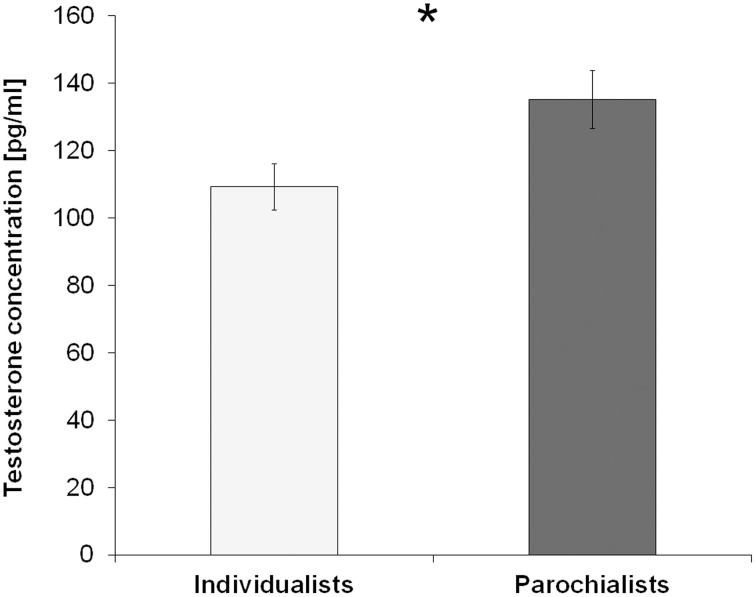
**Testosterone levels predict parochialism**. Subjects displaying a higher tendency for parochial altruism during the competition (i.e., individuals who showed increased cooperation with the ingroup relative to the antagonistic outgroup) had significantly higher testosterone levels than subjects who did not show such a strong ingroup bias (^*^p < 0.05). Error bars indicate standard errors of the mean (SEM).

Considering a possible effect of testosterone on this parochial pattern, a trend for a positive correlation between testosterone and the cooperation rates with the ingroup during the competition was found (Rho = 0.218, *p* = 0.051, one-sided). This relationship was even more pronounced regarding the effect of context as described by the change in cooperation from the competition to the neutral session [i.e., Δcontext (ingroup): Rho = 0.259, *p* = 0.036, one-sided]. Correlations are depicted in Figure 3. In contrast to that, there were no equivalent correlations with ingroup cooperation during the neutral session (Rho = −0.139, *p* = 0.342) or with the overall ingroup cooperation rate across both sessions (Rho = -0.013, *p* = 0.931). To further investigate the effect of testosterone on parochial altruism, we compared the testosterone levels between subjects showing an increased ingroup bias during the competition and subjects that did not differentiate so much between the different teams (i.e., the “parochialists” as compared to the “individualists”). Testosterone levels of the parochialists were significantly higher than those of individualists [*t*_(41)_ = −2.30, *p* = 0.027, *d* = 0.72; testosterone concentrations [mean ± sem] parochialists: 135.10 ± 8.66 pg/ml, individualists: 109.18 ± 6.88 pg/ml]. Figure 4 shows mean testosterone concentrations of both groups. Please also refer to Table 1 to find mean cooperation rates of parochialists and individualists in comparison with those of the whole sample. Interestingly, by following their strategy of increased outgroup hostility and ingroup favoritism parochialists still achieved fewer total payoffs in the competition than individualists [*t*_(41)_ = 5.18, *p* < 0.001, *d* = 1.62; total points [mean ± sem] parochialists: 1647.83 ± 19.83 points, individualists: 1797.00 ± 20.79 points]. This was also reflected by higher overall cooperation rates of parochialists during the competition compared to the individualists (*U* = 56.50, *p* < 0.001; overall cooperation rate [mean ± sem] parochialists: 37.77 ± 3.12 %, individualists: 20.41 ± 3.10 %).

## Discussion

The aim of the present study was to resolve the contradiction regarding the behavioral effects of testosterone (prosocial vs. antisocial) by considering two additional factors, namely group membership and intergroup competition. To test this, male soccer fans played the PD twice, in a neutral and in a group competition context, against counterparts marked as soccer fans of other teams of varying rivalry to the subject's own favorite team. Three major findings emerged: firstly, our results demonstrate the parochial nature of human cooperation with increasing social distance and enmity to the opposing player resulting in decreased cooperation rates. This parochial pattern was observed in both contexts, but was even more prominent during the intergroup competition. The presence of external threat by the competing teams seemed to have intensified parochial tendencies.

Secondly, the present findings suggest testosterone to promote prosocial behavioral tendencies during ingroup interactions since high levels of testosterone were associated with increased cooperation rates with ingroup members during the competitive relative to the neutral context. The fact that an association between testosterone and altruistic behavior could only be found in interactions with the ingroup, suggests a modulating role of testosterone in parochial altruism, which might facilitate group coherence. This thought was supported by the observation that individuals with increased ingroup bias during the competition had significantly higher testosterone levels than individuals who did not differentiate so much between the different groups.

Finally, the observed associations between testosterone and parochial altruism were limited to the group competition or the behavioral adaptation represented by the change in cooperation rates from the competitive as compared to the neutral context. Thus, competition might be a contextual aspect that plays an important role in explaining the effects of testosterone on parochial altruism.

Taken together, the data from the PD complement the previously reported results regarding altruistic punishment in the UG (Diekhof et al., 2014), during which participants displayed the same parochial pattern in their rejection rates (i.e., increasing rejection rates with increasing social distance). Hence, cooperative behavior seems to be affected by parochial tendencies in a similar manner as altruistic punishment. This corresponds to findings from other studies investigating the impact of group membership in social exchange tasks that investigated other types of group membership such as different linguistic language groups of New Guinea (Bernhard et al., 2006) or members of different platoons of the Swiss Army (Goette et al., 2012). Also, the further escalation of ingroup favoritism and outgroup hostility during the competition conforms well to the results of the UG that showed pronounced parochial altruism in a context of intergroup conflict (Diekhof et al., 2014). Important to note, less cooperation with the three outgroups consequently led to higher payoff in the competition than in the neutral context. Nonetheless, the argument that this was due to economically rational behavior rather than parochial altruism is doubtful. First, defection was mainly restricted to outgroups whereas during ingroup interactions altruistic choices for cooperation were observed. Secondly, in the UG outgroup offers were rejected more often during the competition even though in this game this unfavorable treatment involved personal costs (Diekhof et al., 2014). In addition, our results are in line with the observations by Goette et al. (2012), who applied a PD and found that in the context of an intergroup competition between the different Army platoons ingroup cooperation as well as defection in outgroup interactions strongly increased. Hence, the present data fit well into the theoretical framework proposing that intergroup competition may have been the driving force for the co-evolution of parochialism and altruism (Choi and Bowles, 2007). Potentially impeding the interpretation of the present results might be the fact that subjects did not know the exact conversion factor according to which their achieved points were translated into Euros. However, previous studies on decision making during interactions with in- and outsiders have applied all sorts of financial incentives in game theoretic tasks, but nonetheless have all observed prosocial behavior in favor of the own group, which corresponds to our results (e.g., points representing real money in the study by Goette et al., 2012 or even hypothetical endowments as in Campanhã et al., 2011). Further, the sum of the extra group reward was not explicitly mentioned to the subjects, which would allow for an alternative explanation of increased ingroup cooperation during the competition: for all the subjects knew, the extra reward could have as well outweighed the personal loss caused by ingroup cooperation. Nevertheless, when interpreting the subjects' motives to cooperate with ingroup members during the competition it has to be taken into account that only two out of the 50 subjects played completely selfish in the neutral context. This observation is relevant as it suggests that subjects discriminated between the different groups even in the absence of an extra group reward. A possible explanation for this might be that strong emotions of enmity and affiliation between soccer fans dampen the impulse to play economically in the first place (i.e., neutral context). Therefore, it seems plausible that increased ingroup cooperation during the competition indicates parochial altruism rather than a financial strategy.

Considering possible effects of endogenous testosterone, a positive correlation with the change of ingroup cooperation rates from the competitive as compared to the neutral setting emerged. This matches the previous findings from the UG, during which higher salivary testosterone levels were predictive of higher offers to ingroup members (Diekhof et al., 2014). In addition to that, in the UG high testosterone individuals displayed increased outgroup hostility in the form of higher rejection rates toward outgroup proposals during the competitive relative to the neutral context. The PD, however, revealed no specific link between testosterone and outgroup hostility. A possible reason for the absence of an outgroup-directed association between testosterone and aggressive behavior might lie in the specific demands of the PD. While the decision to reject an offer in the UG might in fact indicate an individual's willingness to harm the other player, the decision for no cooperation in the PD might as well result from the intention to protect oneself from exploitation rather than representing an aggressive act against the other player (Rusch, 2014). Thus the PD might not capture outgroup hostility as good as the UG, which could explain the lack of an association between testosterone and outgroup-directed aggression in the present data. In sum, the present results disprove the notion that testosterone is promoting solely antisocial behavior since high levels were associated with increased cooperative behavior in the form of stronger ingroup favoritism. This supports findings from other recent studies reporting prosocial effects of testosterone (Burnham, 2007; Eisenegger et al., 2010; Mehta and Beer, 2010) and points to a more complex role of testosterone in the modulation of human social behavior.

Most importantly, salivary testosterone levels predicted parochial tendencies during the group competition. Testosterone concentrations were higher in subjects displaying a strong ingroup bias than in subjects who treated the teams more equally. Besides the stronger discrimination between the different groups, parochial subjects also won fewer points in the competition than the individualists. This might suggest that besides enhancing ingroup bias, testosterone also facilitates withstanding the impulse to maximize personal payoff for in order to ensure group success. To add further support to this claim we looked again into the data obtained during the UG (Diekhof et al., 2014) and compared behavior in this game between the parochialists and the individualists (as defined here in the present analyses). Matching the findings from the PD, in the UG parochialists showed higher rejection rates in response to unfair offers by antagonistic outgroup members than individualists thereby refraining from the offered points (*U* = 155, *p* = 0.013; rejection rates [mean ± sem] parochialists: 98.26 ± 1.20 %, individualists: 84.00 ± 6.26 %). The observed association between testosterone and parochial altruism in the PD fits well with our previously proposed hypothesis of testosterone as a driving force of intergroup bias. It also conforms well with the “male warrior hypothesis,” which states that specifically males should be more likely to form coalitions and direct aggression toward outgroups during group competitions (Van Vugt et al., 2007; Van Vugt and Park, 2009; McDonald et al., 2012). Since testosterone is the most important sex hormone in males and its role in social behavior has been well described (e.g., Eisenegger et al., 2011), it is reasonable to assume a link between prevalent testosterone levels and parochial altruism in males. The present findings support this assumption by offering evidence for a testosterone-modulated intergroup bias in a group competition context.

Further important to note is that here we report individual differences concerning parochial altruism that were associated with endogenous testosterone levels. However, we cannot exclude possible interferences by other factors, which were not considered in this study. For instance, genetic polymorphisms in the androgen receptor gene might mediate individual behavioral differences that are associated with testosterone. Other open questions that require further research concern influences and interactions by other steroid hormones, such as estrogens, and, especially in this context, if there are comparable effects in females. Against this background, future studies should repeat a similar paradigm and include additional factors to substantiate the observed link between testosterone and parochial altruism.

## Conclusion

This study provides further evidence to the view that testosterone does not only promote antisocial behavioral tendencies, but also facilitates altruism. This was shown here to be specifically the case during an intergroup competition in human males. In this context, testosterone was predictive of parochial altruism (i.e., the favorable treatment of ingroup members, whereas aggression is directed toward the outgroup) and thus was associated with both aggressive and cooperative behavior depending on group membership and competition. The present results are therefore in line with previously stated theories on male coalition building (i.e., “male warrior hypothesis”; Van Vugt et al., 2007) and evolutionary theories on the development of altruism and parochialism (Choi and Bowles, 2007). As a novel finding, they propose testosterone to play a key role in these social mechanisms.

### Conflict of interest statement

The authors declare that the research was conducted in the absence of any commercial or financial relationships that could be construed as a potential conflict of interest.
