# Supplementary material for: Testosterone is associated with cooperation during intergroup competition by enhancing parochial altruism
Source: Front Neurosci. 2015 Jun 12;9:183. doi: 10.3389/fnins.2015.00183 (PMC4464174; doi:10.3389/fnins.2015.00183)
Supplement: Supplementary file 1 [file Presentation1.PDF]

Supplementary Material

Testosterone affects cooperation during intergroup competition by enhancing parochial altruism

Luise Reimers[1]*and Esther K. Diekhof[1]

[1]Neuroendocrinology Unit, Institute for Human Biology, Biocentre Grindle and Zoological Museum, University of Hamburg, Hamburg, Germany

*** Correspondence:** Luise Reimers, Institute for Human Biology, Biocentre Grindle and Zoological Museum, University of Hamburg, Martin-Luther-King Platz 3, D-20146, Hamburg, Germany
luise.reimers@uni-hamburg.de

1. Supplementary information

Written instructions:

Prior to the first session of the Prisoners' Dilemma (PD) subjects received written instructions of the following text:

"The game you will be playing involves two people who have to make a decision. The goal is to gain maximum points.
The situation will be as follows:
Both players are endowed with 20 points at the beginning. You have to decide whether you want to pass on points to the second player (cooperation) or not (no cooperation).
The outcome depends on the decision of the second player.
If both of you decide to cooperate, your points will be doubled and both of you will receive 40 points.
If neither of you cooperates, you will both keep your 20 points.
If you cooperate, but the second player does not, then you will get nothing.
If the second player cooperates, but you don't, then you will receive 60 points.

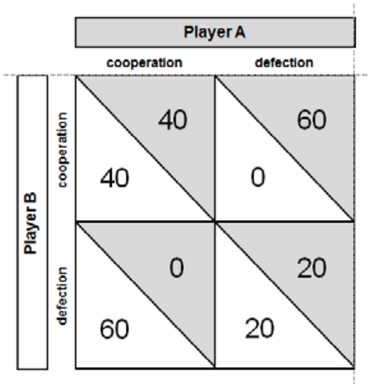

This experiment consists of 40 single rounds, which you are playing against other soccer fans.

Comprehension question:

a) *Player A cooperates and Player B cooperates. How many points does each of them receive?*
b) *Player A cooperates and Player B does not cooperate. How many points does each of them receive?"*

After having completed the first session, subjects were given basically the same instruction again but with a new passage highlighting the competitive context of the second session:

"New in this session:

You can win extra points if your team outperforms all the others by winning most points."
