# Supplementary material for: Testosterone is associated with cooperation during intergroup competition by enhancing parochial altruism
Source: Front Neurosci. 2015 Jun 12;9:183. doi: 10.3389/fnins.2015.00183 (PMC4464174; doi:10.3389/fnins.2015.00183)
Supplement: Supplementary file 2 [file DataSheet1.PDF]

						Cooperation rates [%]: Neutral context				Cooperation rates [%]: Competition context			
ID	Age	Testosterone [pg/ml]	Assay-No.	Ingroup bias competition (variable used for mediansplit)	Mediansplit: Ingroup bias [1=individualists, 2=parochialists, 0=median]	Ingroup	Neutral outgroup	Unknown outgroup	Antagonistic outgroup	Ingroup	Neutral outgroup	Unknown outgroup	Antagonistic outgroup
1	28	119.91	1	70.0	1	30.0	40.0	30.0	30.0	70.0	0.0	0.0	0.0
2	23	210.57	1	100.0	2	10.0	20.0	30.0	0.0	100.0	0.0	0.0	0.0
3	19	139.41	1	100.0	2	50.0	20.0	30.0	10.0	100.0	0.0	0.0	0.0
4	21	117.22	1	80.0	1	90.0	90.0	100.0	10.0	80.0	30.0	10.0	0.0
5	21	153.54	1	90.0	0	80.0	50.0	10.0	0.0	90.0	0.0	0.0	0.0
6	23	207.25	1	100.0	2	100.0	0.0	0.0	0.0	100.0	0.0	0.0	0.0
7	25	115.21	1	40.0	1	70.0	10.0	10.0	20.0	50.0	30.0	10.0	10.0
8	24	101.13	1	90.0	0	50.0	50.0	20.0	0.0	90.0	0.0	0.0	0.0
9	21	107.42	1	80.0	1	100.0	0.0	80.0	0.0	80.0	0.0	60.0	0.0
10	28	108.27	1	100.0	2	100.0	100.0	0.0	0.0	100.0	0.0	0.0	0.0
11	22	130.89	1	100.0	2	100.0	90.0	70.0	20.0	100.0	0.0	100.0	0.0
12	18	106.02	1	100.0	2	0.0	0.0	0.0	0.0	100.0	0.0	0.0	0.0
13	25	147.19	1	100.0	2	100.0	80.0	60.0	0.0	100.0	0.0	0.0	0.0
14	29	67.70	1	90.0	0	100.0	20.0	60.0	0.0	90.0	10.0	10.0	0.0
15	24	104.36	1	100.0	2	100.0	90.0	0.0	10.0	100.0	0.0	0.0	0.0
16	30	57.07	1	0.0	1	20.0	20.0	10.0	20.0	0.0	0.0	0.0	0.0
17	26	163.30	1	100.0	2	100.0	80.0	10.0	0.0	100.0	80.0	0.0	0.0
18	26	120.87	1	100.0	2	10.0	40.0	20.0	30.0	100.0	0.0	0.0	0.0
19	21	90.90	1	60.0	1	100.0	10.0	0.0	0.0	80.0	20.0	10.0	20.0
20	27	76.52	1	80.0	1	40.0	0.0	0.0	0.0	80.0	0.0	0.0	0.0
21	30	159.12	1	100.0	2	40.0	80.0	20.0	10.0	100.0	100.0	0.0	0.0
23	20	170.84	1	100.0	2	100.0	100.0	100.0	20.0	100.0	80.0	50.0	0.0
24	20	138.84	1	100.0	2	0.0	0.0	0.0	0.0	100.0	100.0	0.0	0.0
25	26	130.36	1	80.0	1	30.0	20.0	40.0	0.0	80.0	0.0	0.0	0.0
26	20	132.51	1	0.0	1	30.0	0.0	0.0	0.0	11.0	0.0	10.0	11.0
27	24	130.54	1	60.0	1	80.0	60.0	40.0	0.0	70.0	20.0	0.0	10.0
28	25	80.69	1	100.0	2	100.0	100.0	100.0	100.0	100.0	30.0	30.0	0.0
29	30	165.92	1	90.0	0	40.0	30.0	40.0	20.0	90.0	0.0	10.0	0.0
30	24	123.00	1	100.0	2	60.0	60.0	60.0	0.0	100.0	0.0	0.0	0.0
31	29	91.20	2	100.0	2	50.0	20.0	50.0	0.0	100.0	0.0	0.0	0.0
32	25	72.32	2	40.0	1	100.0	0.0	20.0	0.0	40.0	10.0	10.0	0.0
33	18	225.02	2	100.0	2	0.0	10.0	0.0	10.0	100.0	0.0	0.0	0.0
34	25	61.87	2	30.0	1	30.0	33.0	20.0	30.0	30.0	0.0	0.0	0.0
35	28	117.58	2	80.0	1	100.0	50.0	50.0	0.0	80.0	0.0	0.0	0.0
36	26	112.73	2	100.0	2	80.0	10.0	70.0	20.0	100.0	10.0	0.0	0.0
37	19	157.54	2	20.0	1	20.0	30.0	30.0	20.0	20.0	0.0	0.0	0.0
38	23	100.21	2	100.0	2	100.0	100.0	20.0	0.0	100.0	100.0	0.0	0.0
39	24	77.99	2	0.0	1	0.0	0.0	0.0	0.0	0.0	0.0	0.0	0.0
40	21	122.01	2	90.0	0	30.0	40.0	30.0	0.0	90.0	0.0	0.0	0.0
41	32	112.73	2	60.0	1	30.0	60.0	20.0	30.0	60.0	10.0	10.0	0.0
42	26	109.74	2	100.0	2	40.0	10.0	30.0	10.0	100.0	0.0	0.0	0.0
43	20	130.40	2	0.0	1	40.0	30.0	0.0	0.0	0.0	0.0	0.0	0.0
44	29	174.24	2	0.0	1	0.0	0.0	40.0	0.0	0.0	0.0	0.0	0.0
45	29	177.54	2	100.0	2	100.0	100.0	0.0	0.0	100.0	90.0	20.0	0.0
46	27	82.57	2	100.0	2	100.0	70.0	20.0	0.0	100.0	0.0	20.0	0.0
47	27	114.03	2	0.0	1	0.0	0.0	0.0	0.0	0.0	0.0	0.0	0.0
48	24	87.25	2	80.0	1	10.0	0.0	10.0	30.0	80.0	0.0	0.0	0.0
49	26	101.77	2	90.0	0	90.0	50.0	100.0	10.0	90.0	30.0	80.0	0.0
50	25	97.17	2	100.0	2	100.0	80.0	50.0	50.0	100.0	0.0	10.0	0.0

			Delta context: cooperation rate [%] competition - neutral context										
ID	Testosterone [pg/ml]	Mediansplit: Ingroup bias [1=individualists, 2=parochialists, 0=median]	Delta ingroup	Delta neutral outgroup	Delta unknown outgroup	Delta antagonistic outgroup	overall cooperation rate [%]: neutral context	overall cooperation rate [%]: competition	overall cooperation rate [%]: both sessions	Total points: neutral context	Total points: competition	Total points: both sessions
1	119.91	1	40.0	-40.0	-30.0	-30.0	32.5	17.5	25.00	1640	1760	3400
2	210.57	2	90.0	-20.0	-30.0	0.0	15.0	25.0	20.00	1780	1700	3480
3	139.41	2	50.0	-20.0	-30.0	-10.0	27.5	25.0	26.25	1700	1720	3420
4	117.22	1	-10.0	-60.0	-90.0	-10.0	72.5	30.0	51.25	1340	1680	3020
5	153.54	0	10.0	-50.0	-10.0	0.0	35.0	22.5	28.75	1640	1740	3380
6	207.25	2	0.0	0.0	0.0	0.0	25.0	25.0	25.00	1720	1720	3440
7	115.21	1	-20.0	20.0	0.0	-10.0	27.5	25.0	26.25	1700	1720	3420
8	101.13	0	40.0	-50.0	-20.0	0.0	30.0	22.5	26.25	1680	1740	3420
9	107.42	1	-20.0	0.0	-20.0	0.0	45.0	35.0	40.00	1560	1640	3200
10	108.27	2	0.0	-100.0	0.0	0.0	50.0	25.0	37.50	1520	1720	3240
11	130.89	2	0.0	-90.0	30.0	-20.0	70.0	50.0	60.00	1360	1520	2880
12	106.02	2	100.0	0.0	0.0	0.0	0.0	25.0	12.50	1920	1720	3640
13	147.19	2	0.0	-80.0	-60.0	0.0	60.0	25.0	42.50	1440	1720	3160
14	67.70	0	-10.0	-10.0	-50.0	0.0	45.0	27.5	36.25	1560	1700	3260
15	104.36	2	0.0	-90.0	0.0	-10.0	50.0	25.0	37.50	1520	1720	3240
16	57.07	1	-20.0	-20.0	-10.0	-20.0	17.5	0.0	8.75	1780	1920	3700
17	163.30	2	0.0	0.0	-10.0	0.0	47.5	45.0	46.25	1540	1560	3100
18	120.87	2	90.0	-40.0	-20.0	-30.0	25.0	25.0	25.00	1720	1720	3440
19	90.90	1	-20.0	10.0	10.0	20.0	27.5	32.5	30.00	1700	1660	3360
20	76.52	1	40.0	0.0	0.0	0.0	10.0	20.0	15.00	1840	1760	3600
21	159.12	2	60.0	20.0	-20.0	-10.0	37.5	50.0	43.75	1620	1520	3140
23	170.84	2	0.0	-20.0	-50.0	-20.0	80.0	57.5	68.75	1280	1460	2740
24	138.84	2	100.0	100.0	0.0	0.0	0.0	50.0	25.00	1920	1520	3440
25	130.36	1	50.0	-20.0	-40.0	0.0	22.5	20.0	21.25	1740	1760	3500
26	132.51	1	-19.0	0.0	10.0	11.0	7.5	8.0	7.75	1860	1820	3680
27	130.54	1	-10.0	-40.0	-40.0	10.0	45.0	25.0	35.00	1560	1720	3280
28	80.69	2	0.0	-70.0	-70.0	-100.0	100.0	40.0	70.00	1120	1600	2720
29	165.92	0	50.0	-30.0	-30.0	-20.0	32.5	25.0	28.75	1660	1720	3380
30	123.00	2	40.0	-60.0	-60.0	0.0	45.0	25.0	35.00	1560	1720	3280
31	91.20	2	50.0	-20.0	-50.0	0.0	30.0	25.0	27.50	1680	1720	3400
32	72.32	1	-60.0	10.0	-10.0	0.0	30.0	15.0	22.50	1680	1800	3480
33	225.02	2	100.0	-10.0	0.0	-10.0	5.0	25.0	15.00	1880	1720	3600
34	61.87	1	0.0	-33.0	-20.0	-30.0	28.3	7.5	17.88	1680	1860	3540
35	117.58	1	-20.0	-50.0	-50.0	0.0	50.0	20.0	35.00	1520	1760	3280
36	112.73	2	20.0	0.0	-70.0	-20.0	45.0	27.5	36.25	1560	1700	3260
37	157.54	1	0.0	-30.0	-30.0	-20.0	25.0	5.0	15.00	1720	1880	3600
38	100.21	2	0.0	0.0	-20.0	0.0	55.0	50.0	52.50	1480	1520	3000
39	77.99	1	0.0	0.0	0.0	0.0	0.0	0.0	0.00	1920	1920	3840
40	122.01	0	60.0	-40.0	-30.0	0.0	25.0	22.5	23.75	1720	1740	3460
41	112.73	1	30.0	-50.0	-10.0	-30.0	35.0	20.0	27.50	1640	1760	3400
42	109.74	2	60.0	-10.0	-30.0	-10.0	22.5	25.0	23.75	1740	1720	3460
43	130.40	1	-40.0	-30.0	0.0	0.0	17.5	0.0	8.75	1780	1920	3700
44	174.24	1	0.0	0.0	-40.0	0.0	10.0	0.0	5.00	1840	1920	3760
45	177.54	2	0.0	-10.0	20.0	0.0	50.0	52.5	51.25	1520	1500	3020
46	82.57	2	0.0	-70.0	0.0	0.0	47.5	30.0	38.75	1540	1680	3220
47	114.03	1	0.0	0.0	0.0	0.0	0.0	0.0	0.00	1920	1920	3840
48	87.25	1	70.0	0.0	-10.0	-30.0	12.5	20.0	16.25	1820	1760	3580
49	101.77	0	0.0	-20.0	-20.0	-10.0	62.5	50.0	56.25	1420	1520	2940
50	97.17	2	0.0	-80.0	-40.0	-50.0	70.0	27.5	48.75	1360	1700	3060
